# Supplementary material for: JNK pathway suppression mediates insensitivity to combination endocrine therapy and CDK4/6 inhibition in ER+ breast cancer
Source: J Exp Clin Cancer Res. 2025 Aug 19;44:244. doi: 10.1186/s13046-025-03466-9 (PMC12363127; doi:10.1186/s13046-025-03466-9)

Supplementary Figure 1


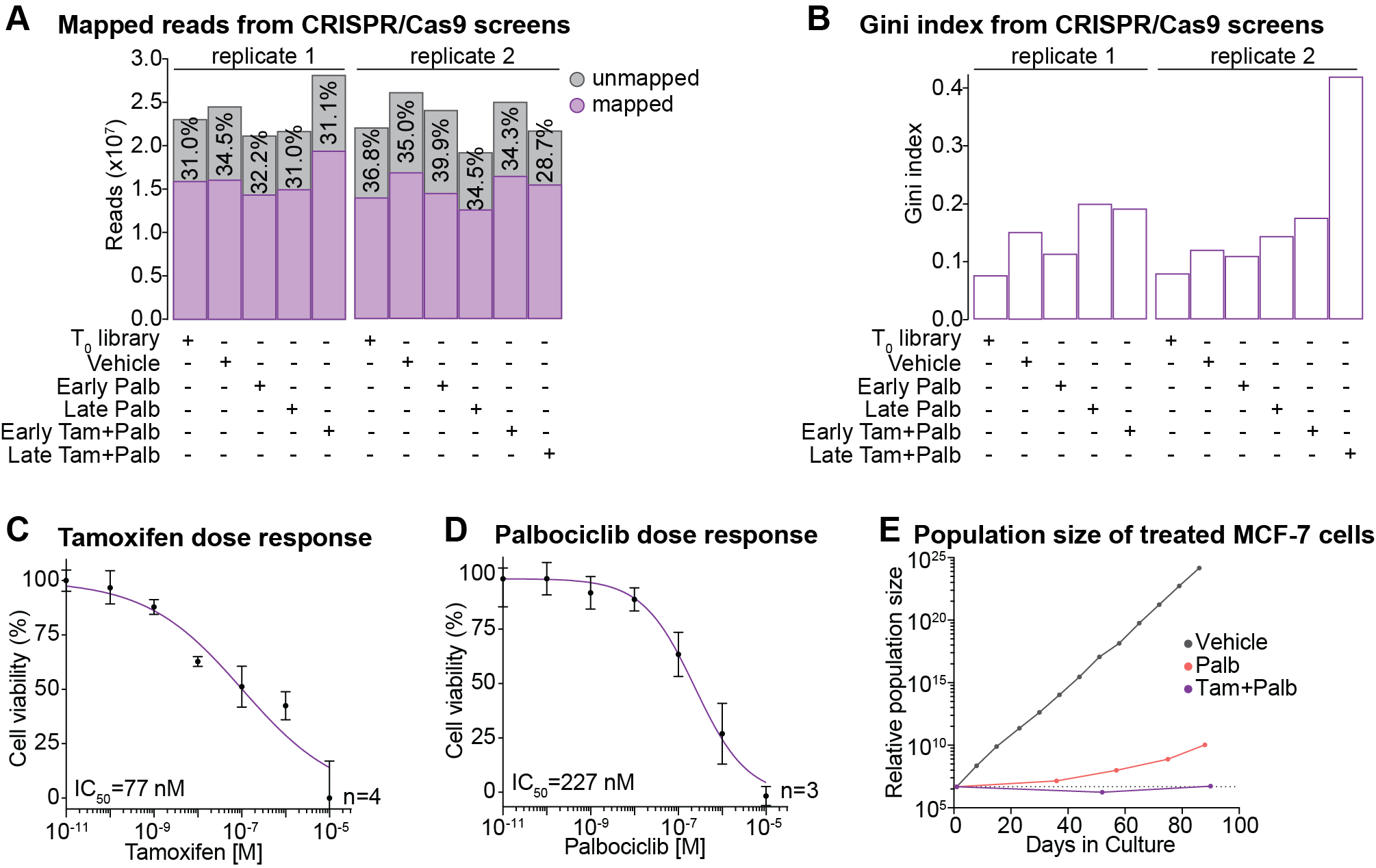


Supplementary Figure 2


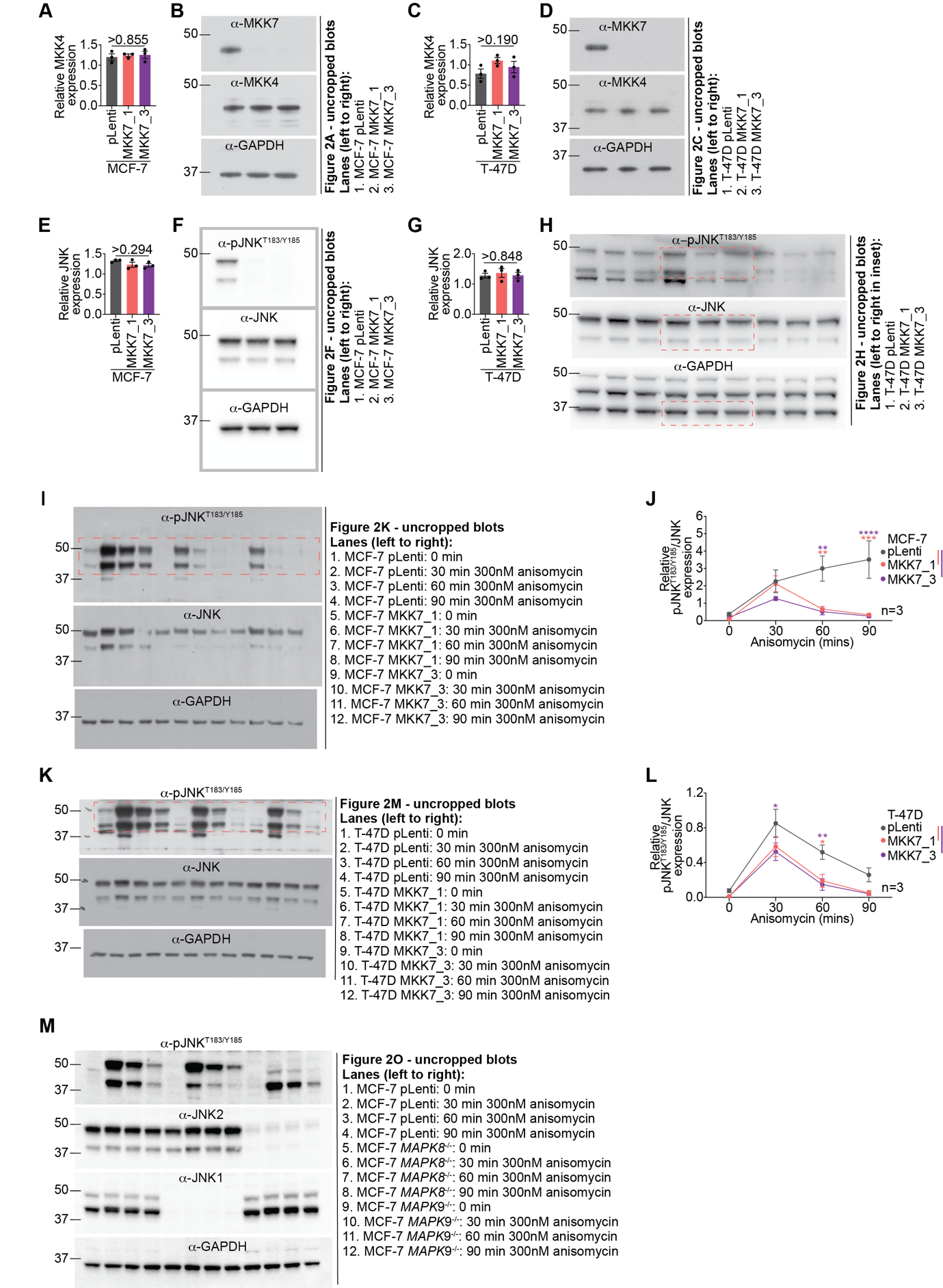


Supplementary Figure 3


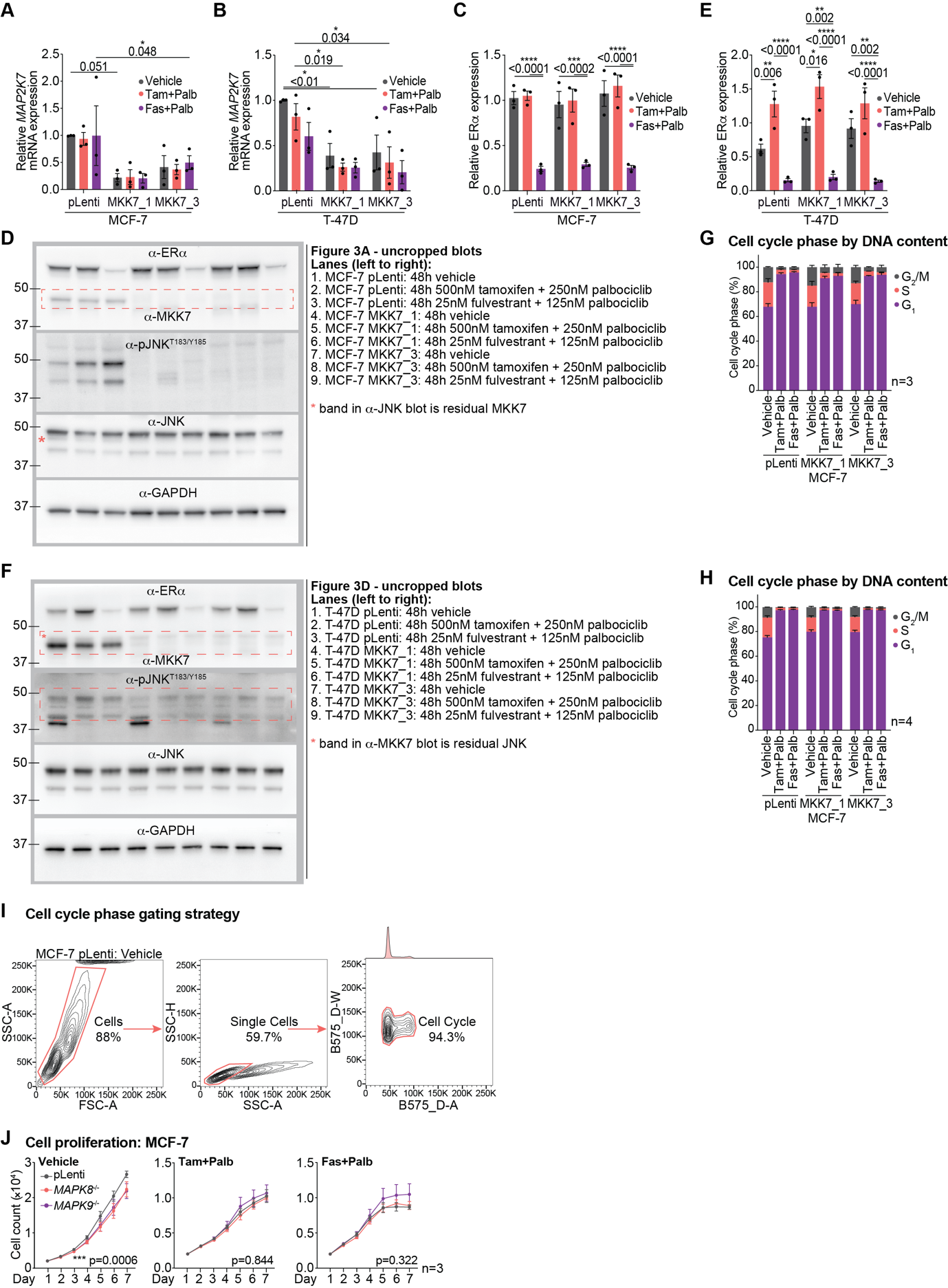


Supplementary Figure 4


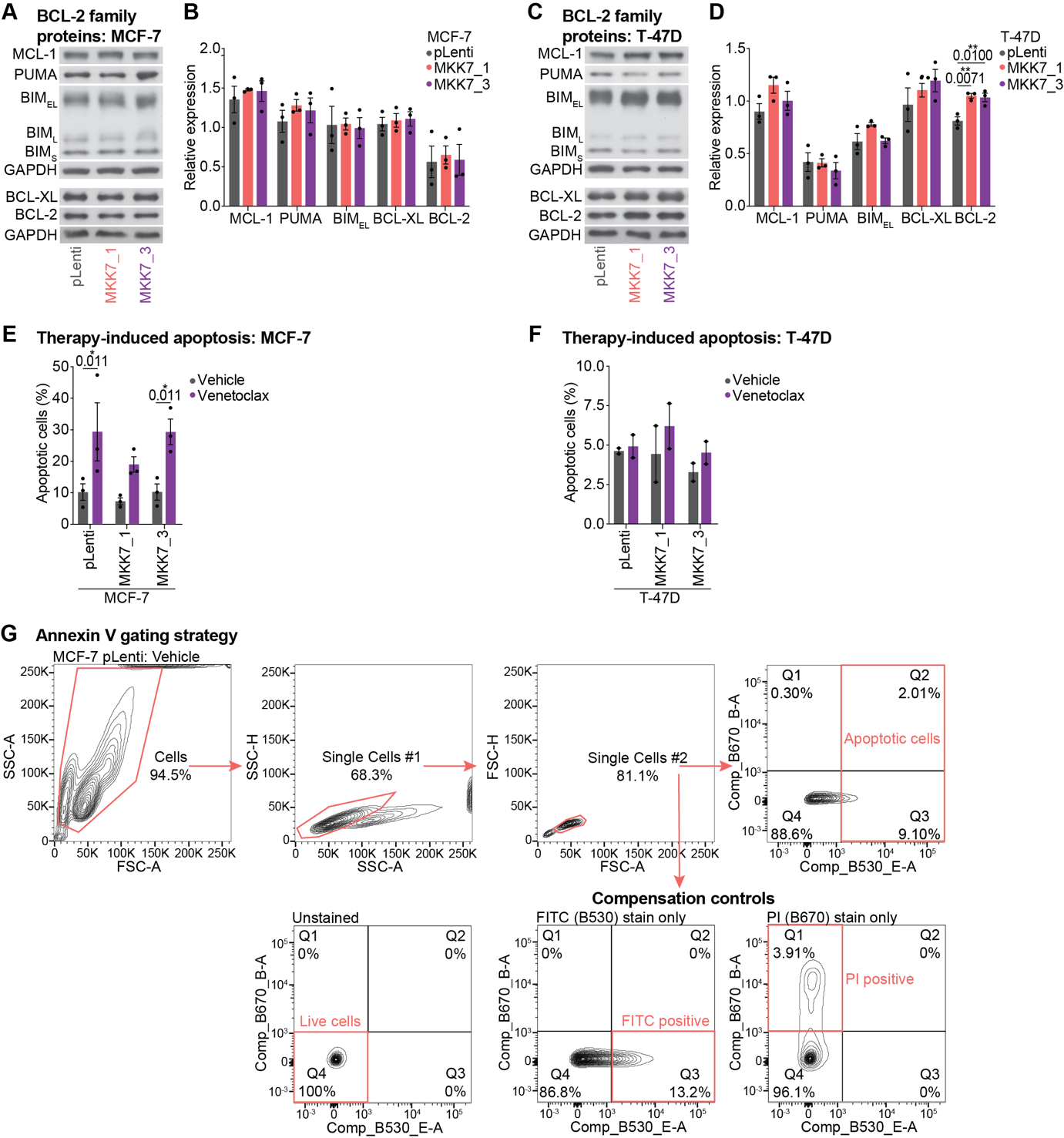


Supplementary Figure 5


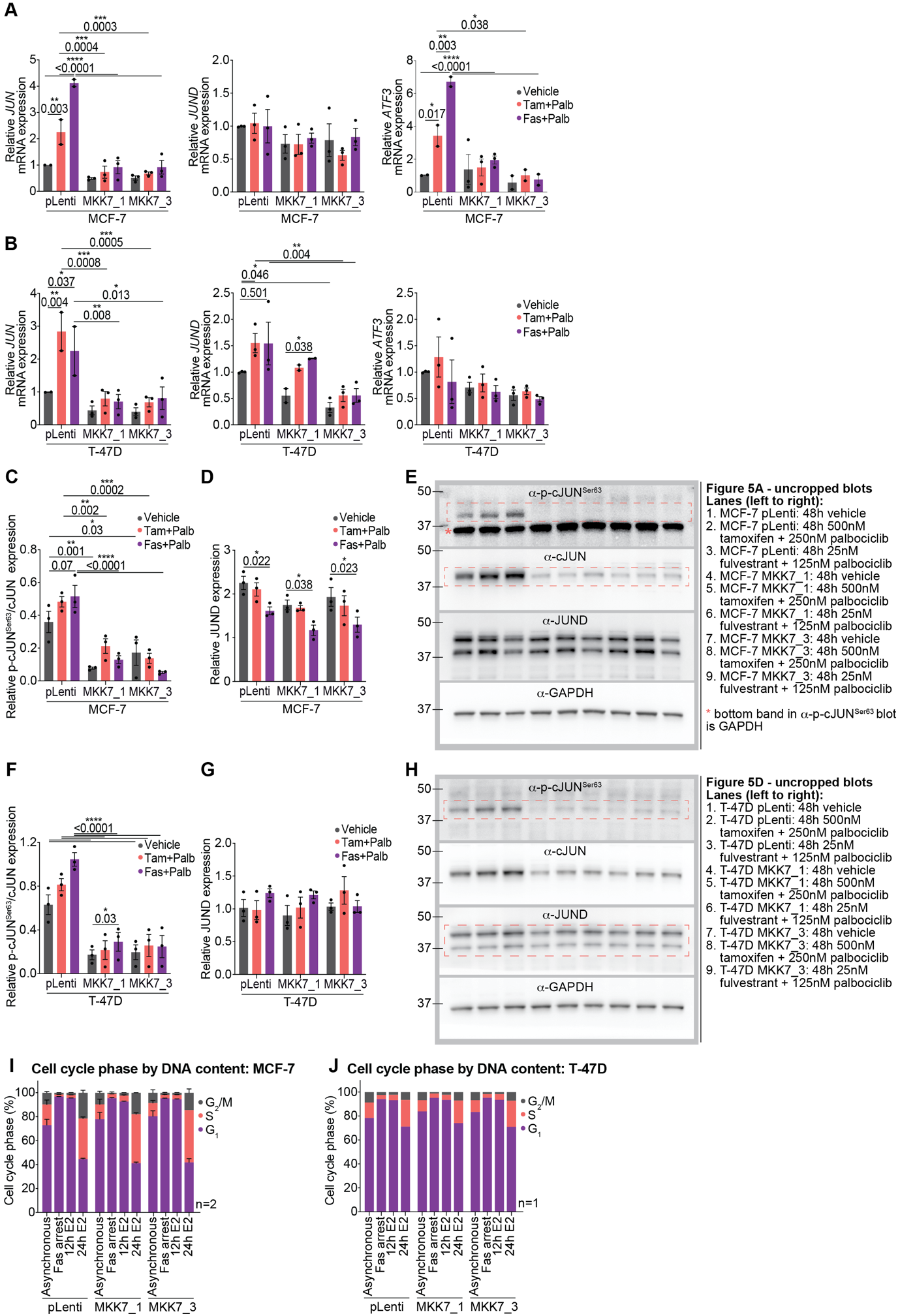


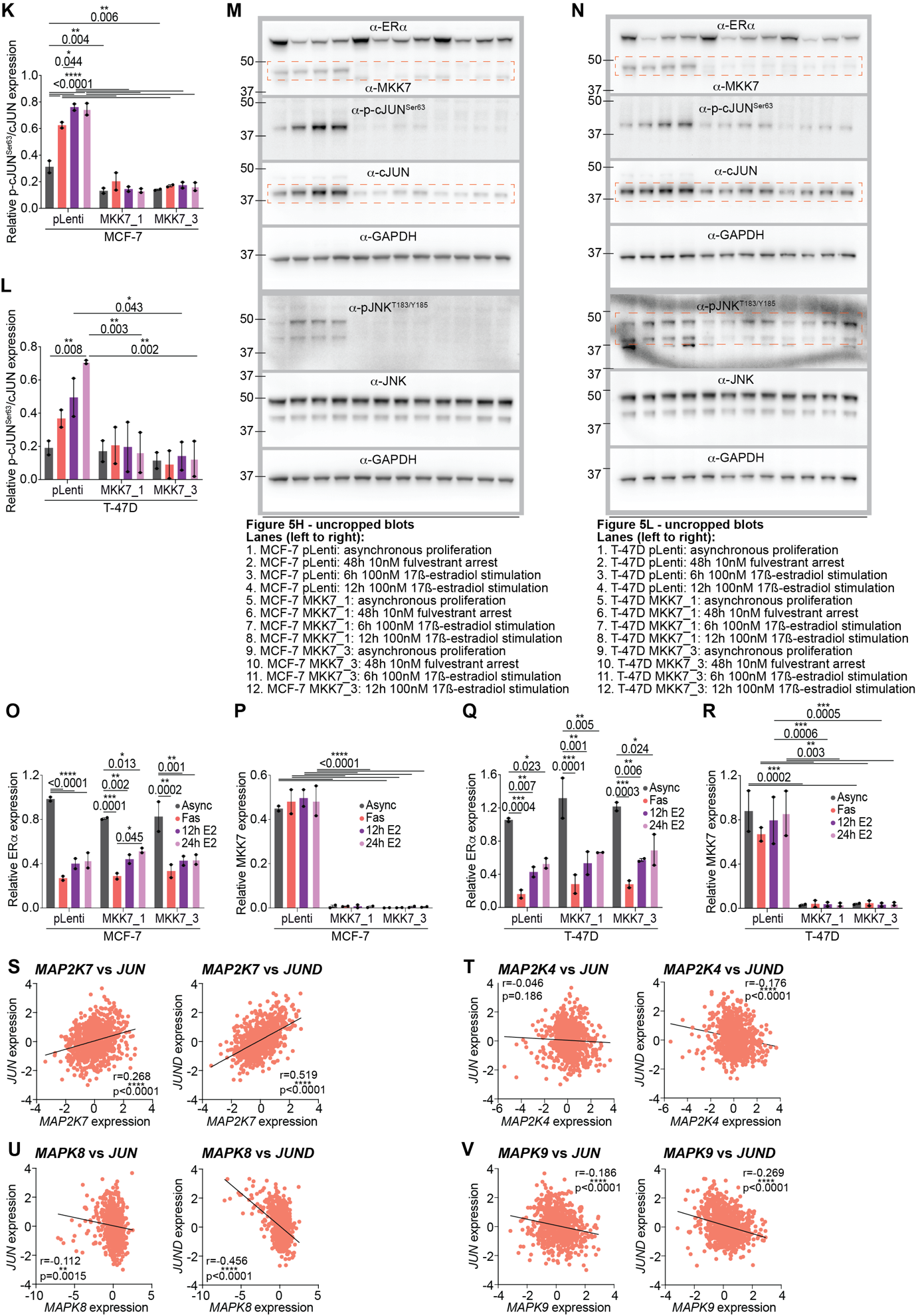


Supplementary Figure 6


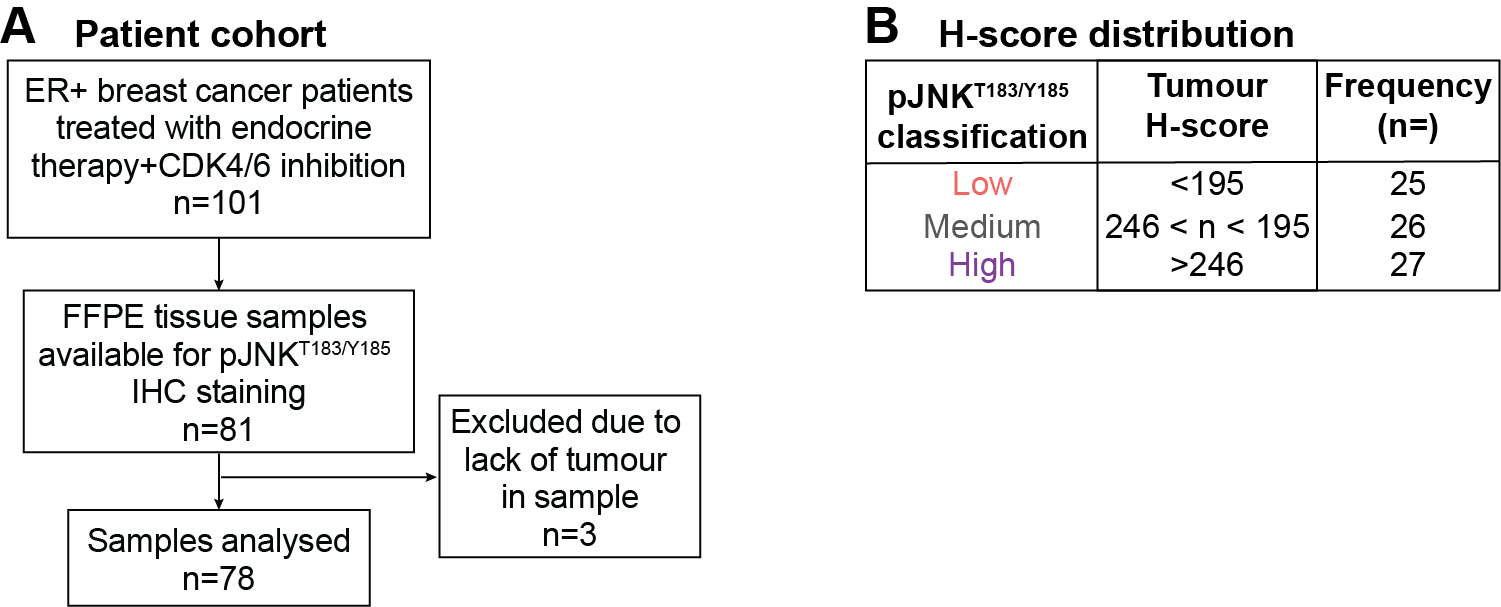

Supplement: Supplementary file 5 — Supplementary Fig. 1: Quality control from CRISPR/Cas9 screens and MCF-7 dose response curves (A) Total reads, both mapped and unmapped, across 2 biological CRISPR/Cas9 screens. (B) Gini index (a measure of inequality, with 1 being the most unequal) compared to the starting cell population (T0) and between replicates. Data in (A and B) were analysed using MAGeCK-VISPR [19]. (C) Dose response curve of MCF-7 cells treated for 5 days with tamoxifen. IC50 values were determined by log transforming, normalising, and plotting data with a nonlinear fit of least square analysis. Experiment performed in quadruplicate. (D) Dose response curve of MCF-7 cells treated for 5 days with palbociclib. IC50 values were determined by log transforming, normalising, and plotting data with a nonlinear fit of least square analysis. Experiment performed in triplicate. (E) Population size of MCF-7 cells treated for up to 90 days with 500 nM palbociclib (palb), 500 nM tamoxifen + 250 nM palbociclib (tam + palb), or vehicle (tetrahydrofuran). Dotted line indicates starting cell number. Supplementary Fig. 2: Effect of MAP2K7 knockout on MKK4 and JNK expression (A) Quantitation of MKK4 protein expression in MCF-7 pLenti and MAP2K7−/− (MKK7_1 and MKK7_3) cells by densitometry. Band intensity normalised to respective GAPDH. Data analysed by one-way ANOVA with Tukey’s multiple comparisons test. (B) Uncropped Western blots for Fig. 2A. Polyvinylidene fluoride (PVDF) membranes were sliced into smaller sections to incubate with primary antibodies to allow accurate comparison of protein expression within a single sample. Full sections of the sliced membranes from each cropped Western blot are shown. (C) Quantitation of MKK4 protein expression in T-47D pLenti and MAP2K7−/− (MKK7_1 and MKK7_3) cells by densitometry. Band intensity normalised to respective GAPDH. Data analysed by one-way ANOVA with Tukey’s multiple comparisons test. (D) Uncropped Western blots for Fig. 2C. PVDF membranes were sliced into [file 13046_2025_3466_MOESM5_ESM.docx]
